# On the Formation of the Buffy Coat of the Blood

**Published:** 1845-04

**Authors:** George Gulliver

**Affiliations:** Surgeon in the Royal Regiment of Horse Guards


*On the formation of the Buffy Coat of the Blood.*—By George Gulliver, F.R.S., Surgeon in the Royal Regiment of Horse Guards.—After noticing the well known fact, that the red corpuscles will sink much faster in the entire blood, while the buffy coat is forming, than they will do in the serum alone, the author remarks that two of the most eminent authorities on the physiology of the blood, Mr. Hewson and Dr. Davy, concluded that the rapid sinking of the corpuscles on which the buffy coat depends, is due to an attenuation of the liquor sanguinis. On the contrary, Professor Hermann, Nasse, and Mr. Jones, attribute the quick sinking of the corpuscles to their increased aggregation in inflammatory blood.—*British and Foreign Medical Review.*

Finding that the blood of the horse generally affords a buffy surface, measuring as much perpendiculary as the lower red part of the blood clot, the author made many experiments on the sinking of the red corpuscles in the liquor sanguinis in the serum, and in these fluids variously altered in viscidity and specific gravity. The experiments are particulary detailed, in order that they may be easily repeated, and that no errors may arise from the different effects which Mr. Prater has shewn may be produced by different quantities of the same substance.

The author is of opinion, that if we admit the sinking of the red corpuscles affords an accurate test of the consistency of the liquor sanguinis, we must also admit the improbability that the liquor sanguinis, becomes *thinner some minutes after the blood has been received in a vase,* at which time the falling of the red corpuscles is most rapid.

The author asks whether the well-known utility of saline medicines in inflammation may not be explained by their effects in preventing or destroying the aggregation of the red corpuscles and in preventing or lessening the buffy or inflammatory condition of the blood.

The author arranges some of his conclusions as follows:—

First—There is a remarkable acceleration, after a few minutes, in the ratio with which the blood corpuscles sink in the liquor sanguinis.

Second.—This acceleration may be increased by increasing the aggregation of the corpuscles; and prevented or removed, by preventing or destroying the aggregation of the corpuscles.

Third.—The sinking of the corpuscles is slower in blood thinned by weak saline solutions, than when mucilage is added with the salt.

Fourth.—The sinking of the corpuscles may be slower in serum artificially made thinner and lighter, than in serum artificially made thinner and heavier.

Fifth.—In the cruor of horses' blood the corpuscles are more aggregated, and with more appearance of agglutination between the corpuscles, than in very buffy human blood.

Sixth.—There may be a buffy coat, or only a comparatively thin one, on the blood of the horse, when the blood has been made thinner and its coagulation retarded.

Seventh.—The corpuscles of the horse sink much quicker in his serum than the corpuscles of man do in his.

Eighth.—Increasing the proportion of corpuscles in the blood hastens coagulation, and prevents or diminishes the formation of the buffy coat, more than increasing the serum only.—*Lond. Lancet.*

